# Exploring relationships between abnormal within-network functional connectivity, preoperative brain tumor variables, and neuropsychological test scores

**DOI:** 10.1093/noajnl/vdag084

**Published:** 2026-04-03

**Authors:** Bryce J Laurin, Randall Treffy, Christina Feller, Joshua Wilder, Krish Vasudev, Nicholas Schulz, Léon Taquet, Melissa Lancaster, Alissa Butts, Timothy F Boerger, Brian D Schmit, Max O Krucoff

**Affiliations:** School of Medicine, Medical College of Wisconsin, Milwaukee, Wisconsin, USA; Department of Psychology, Marquette University, Milwaukee, Wisconsin, USA; Undergraduate Studies, Washington University, St. Louis, Missouri, USA; Department of Neurosurgery, Medical College of Wisconsin, Milwaukee, Wisconsin, USA; Department of Neurology, Division of Neuropsychology, Medical College of Wisconsin, Milwaukee, Wisconsin, USA; Department of Biomedical Engineering, Marquette University and Medical College of Wisconsin, Milwaukee, Wisconsin, USA; Department of Biomedical Engineering, Marquette University and Medical College of Wisconsin, Milwaukee, Wisconsin, USA

**Keywords:** brain network, cognitive outcomes, connectomics, glioma, neurosurgery

## Abstract

**Objective:**

Neuropsychological symptoms in people with brain tumors are common; however, they are often incompletely explained by tumor variables (such as anatomical location, size, or grade) and may be more directly related to changes in large-scale functional network connectivity. Here we examine these relationships.

**Methods:**

Fifty-one participants underwent pre-operative resting-state functional MRIs and three neuropsychological tests—Trail Making Test-Part-B (TMT-B), WAIS-IV Digit-Span Sequencing (WAIS-DS), and Controlled Oral Word Association Test (COWAT). Within-network functional connectivity of the central executive (CEN), default mode (DMN), language (LANG), and salience (SN) networks were compared to healthy controls. Spearman correlations (ρ) were calculated between neuropsychological z-scores, abnormal (>2 standard deviations from control means) within-network connectivity, and tumor variables while controlling for multiple comparisons. Exploratory, statistical mediation analyses then evaluated if relevant tumor variables affected neuropsychological performance via changes in functional connectivity.

**Results:**

Significant correlations included: (1) WAIS-DS performance to lesional-SN (*ρ* = 0.53, *P* = 0.006), lesional-CEN (*ρ* = 0.42, *P* = 0.023), and right-SN (*ρ* = 0.42, *P* = 0.023) connectivity; (2) COWAT performance to right-SN (*ρ* = 0.50, *P* = 0.012), lesional-SN connectivity (*ρ* = 0.45, *P* =0 .017), and lesion laterality (*ρ* =  0.47, *P* = 0.017); and (3) TMT-B to lesional-LANG (*ρ* =  0.46, *P* =0 .017), right-CEN (*ρ* = 0.45, *P* = 0.017), and bilateral-LANG (*ρ* =  0.42, *P* = 0.024) connectivity. Mediation analyses revealed the following effects: (1) lesion laterality on TMT-B was fully mediated via right-CEN connectivity (path a*b; *β* = 0.696 [0.13, 1.419]); (2) IDH-status on WAIS-DS was fully mediated via lesional-CEN connectivity (path a*b; *β* = 0.251 [0.015, 0.588]); and (3) lesion laterality on COWAT was partially mediated via right-SN connectivity (path a*b; *β* = 0.333 [0.004-0.72]).

**Conclusions:**

Our data support the hypothesis that functional network connectivity may explain some neuropsychological heterogeneity across otherwise anatomically and oncologically similar cases. Notably, more abnormal connectivity correlated with better performance, suggesting compensatory reorganization may be at least partially responsible.

Key Points

- Abnormal, within-network, resting state functional connectivity correlated with multiple neuropsychological test scores moreso than many tumor variables
- Tumors impacted neuropsychological scores both directly (i.e., not mediated by connectivity) and indirectly (i.e., mediated by connectivity).
- More abnormal within-network connectivity correlated with better neuropsychological test scores, suggesting that compensatory mechanisms may explain some phenotypical heterogeneity across otherwise oncologically and anatomically similar lesions.

Importance of the Study

Neuropsychological deficits in people with brain tumors are common but not fully explained by tumor characteristics such as size, location, or grade. Our study examined the relationship between neuropsychological performance and abnormal within-network functional connectivity within the central executive (CEN), salience (SN), default mode (DMN), and language (LANG) networks. We found that more abnormal connectivity was associated with better neuropsychological performance. Further, abnormal connectivity was more strongly correlated with neuropsychological test scores than some tumor variables. Connectivity also mediated the relationships between tumor variables and neuropsychological test scores. These findings suggest compensatory reorganization may be at least partially responsible for maintaining certain cognitive functions, and it highlights functional-anatomical relationships that could be targets for future neuromodulation studies.

Planning neurosurgical procedures for intra-axial tumors requires carefully balancing oncological goals of aggressive tumor resection with functional goals of preserving normal brain.^1^ Primary motor and language areas can be localized and preserved using various well-described techniques, including preoperative structural and functional imaging and intraoperative stimulation mapping.^2^^,^^3^ In contrast, neuropsychological functions such as executive control and emotional-behavioral regulation are more widely distributed, making them more difficult to localize, predict, and protect.^4–7^ Unfortunately, deficits in these functional domains can have just as profound an impact on quality of life and survival as motor and speech problems.^8^ In this context, there is increasing interest in better understanding the cognitive footprint of potential surgical approaches by applying newer models of neurological function developed through recent neuroscientific advances, such as the human connectome project (HCP).^9^^,^^10^ In other words, we (and others) hypothesize that direct assessments of brain network connectivity may better explain the array of neuropsychological and cognitive symptoms seen in brain tumor patients when compared to classical variables of anatomical location and tumor characteristics.^11^

*Structural* brain network connectivity can be assessed radiographically through diffusion-weighted tractography which traces the diffusion of water molecules through white matter tracts to provide an approximation of physical connections between brain areas.^12^ While fundamental, the mere spatial organization of these pathways is insufficient in explaining the full array of the brain’s functional activity, and the relationships between brain structure and function can be highly nonlinear.^13^ Consequently, recent efforts have been directed toward defining an additional *functional* connectome metrics using resting-state functional magnetic resonance imaging (rs-fMRI). Functional-MRI measures blood oxygenation level dependent (BOLD) signals delivered over time to each MRI voxel, representing summated local neuronal activity.^14^^,^^15^ Areas of the brain that have highly correlated BOLD signal-time series patterns can be sorted into a set of stable networks that appear to subserve many functional domains of human behavior.^15–19^ For example, one foundational study by Yeo et al. stably divides the brain into a minimum of 7 discrete, stable networks^20^, which now has been independently replicated and verified repeatedly.^21–24^ Although there is no generally agreed upon set of terminology for these networks, they are commonly called the central executive (CEN), default mode (DMN), dorsal attention (DAN), limbic/paralimbic (Limbic), salience (SN), sensorimotor (SMN), and visual (VN) networks.^20^ Notably, a language network (LANG) can be extracted from the other 7 networks (primarily DMN) to make up an 8-network model.

The SN, CEN, and DMN are thought to play strong roles in almost all cognitive functions.^25^ The DMN is active during resting states and is associated with self-directed (i.e., “internal”) thoughts.^25^ Opposing the DMN is the CEN, which actively uses inputs from other networks and working memory to maintain concentration during goal-directed (i.e., “external”) behavior and decision-making.^25^ The SN coordinates activity between the DMN and CEN and receives information about the outside world from viscerosensory cortices to determine which, if any, of these stimuli need to be brought to conscious attention (i.e., what is “salient”).^26^ The SN is thought to alter the brain’s dominant state between concentration on oneself, mediated by the DMN, and task-based attention on external stimuli, mediated by the CEN.^25^ These three networks function together in higher-order processing of internal and external stimuli to guide behavioral strategies, and dysfunction within this system is thought to contribute dose-dependently to psychiatric disorders with substantial disruption linked to severe conditions like schizophrenia and mild dysfunction associated with disorders such as anxiety.^27^ However, whether these findings translate to patients with organic lesions, such as brain tumors, is just beginning to be examined.

Consistent with this theoretical framework, here we explored the hypothesis that changes in cognition caused by invasive brain tumors might work most directly through changes in the functional connectome (Figure 1). To this end, we assessed rs-fMRI and neuropsychological data from 51 consecutive preoperative people with intra-axial lesions to examine whether abnormal functional connectivity in the CEN, DMN, LANG (Supplementary Figure S1), and SN might correlate more strongly with presenting neuropsychological symptoms (i.e., clinical phenotype) than classical tumor variables such as size, location, and World Health Organization (WHO) grade.

**Figure 1.**
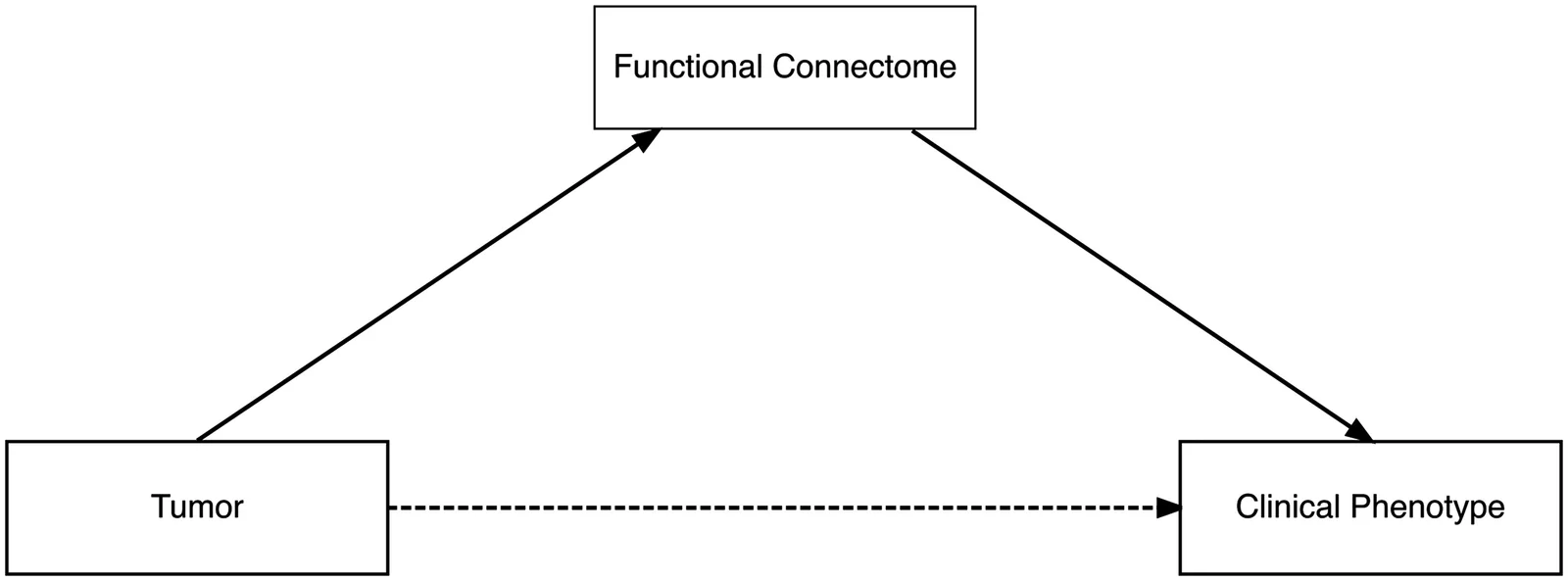
Conceptual framework for how tumors may affect cognition and behavior. The solid arrows represent an indirect pathway, proposed here, where tumors modulate clinical phenotype by altering the functional connectome. Alternatively, the dashed arrow represents a direct pathway where tumors may affect clinical phenotype directly.

## Methods

### Participants

We analyzed data from 51 consecutive people with intra-axial brain tumors who had completed rs-fMRI imaging and a neuropsychological battery prior to surgery. Patient age, race, sex, handedness, preoperative Karnofsky Performance Score (KPS), and the time between rs-fMRI, neuropsychological testing, and surgery were obtained (Table 1).

**Table 1.** Summary of demographic data, neuropsychological test performance (reported as mean z-score ± standard deviation), clinical timing metrics, and tumor variables for the patient cohort (N = 51)

Demographic Data	Mean or #
Age	56.7 (±14.3)
Sex (M/F)	30/21
KPS	90 (±10)
Handedness (L/R)	7/44
***Neuropsychologic Test Results ***	
TMT-B	−1.71 (±2.08)
WAIS-DS	−0.253 (±0.804)
COWAT	−0.656 (±1.49)
***Clinical Timing Metrics ***	
Days from test to rs-fMRI	3.47 (±7.77)
Days from test to surgery	8.75 (±14.57)
***Tumor Variables***	
Glioma	43
Metastasis	8
IDH status (wt/mut)	28/14
MGMT (methylated/non-methylated)	25/17
Grade I	1
Grade II	10
Grade III	1
Grade IV	31
Tumor volume (cm^3^)	20.6 (±22.2)
Lesion laterality (L/R)	28/23
Frontal	19
Temporal	16
Parietal	12
Occipital	3
Cerebellar	1

Karnofsky Performance Score (KPS), Trail Making Test (Part B, TMT-B), Wechsler Adult Intelligence Scale (WAIS)-4 Digit Span (WAIS-DS), Controlled Oral Word Association Test (COWAT), Wild-type (wt), mutant (mut).

### Neuropsychological Testing

We used three well-established tests with published age-controlled norms to assess critical components of attention, working memory, language, and executive function: Trail Making Test-Part B (TMT-B), Wechsler Adult Intelligence Scale Fourth Edition Digit Span Sequencing (WAIS-DS), and the Controlled Oral Word Association Tests (COWAT). TMT-B is a timed test that isolates higher planning and executive functioning through switching between numbers and letters.^28^ The WAIS-DS is a test of attention and working memory that requires participants to order a set of digits forward, backward, then sequenced on separate trials. The COWAT is a word retrieval test that requires the participant to generate words that start with a certain letter within a certain amount of time^29^^,^^30^ (Supplementary Table S1). Raw scores were converted to z-scores using age and education level-matched normative data for each test.^31–33^ While participants overall exhibited lower baseline performance as reflected by negative mean z-scores, the distribution of these scores remained approximately normal, indicating preserved variability in cognitive performance across the cohort.

### MRI Data Acquisition

All participants underwent a preoperative MRI with sequences including 3D/volumetric T1 with-and-without contrast, T2, and Fluid-Attenuated Inversion Recovery (FLAIR) anatomical scans for characterization of tumor location, size, edema, and mass effect. Resting state-fMRI scans were obtained on a GE SIGNA (GE Healthcare, Milwaukee, Wisconsin) Architect 3T magnet with a 48-channel head coil (TE = 30/TR = 2418-3154 ms, matrix = 128 × 128, voxel size = 3 × 3×3 mm, flip angle = 90, slice spacing = 3mm, Acceleration factor = 2, 8.06-10.5 minutes). Participants were instructed to rest quietly with their eyes open, looking at a fixation cross, and to let their minds wander without falling asleep.

### Functional Connectome Generation

**Figure 2.**
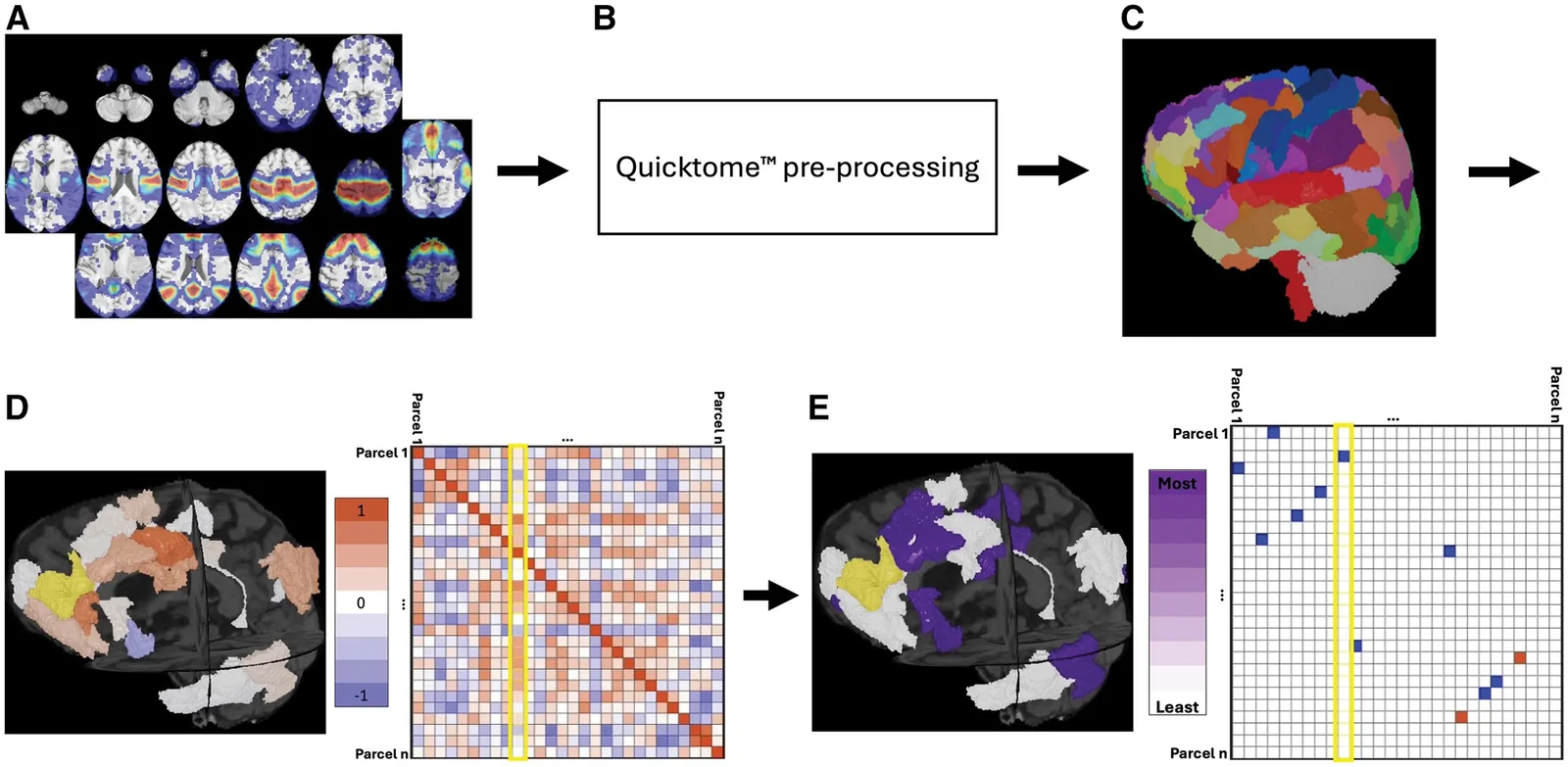
Image processing workflow. Resting state-fMRI data (A; BOLD signal time series) is uploaded and pre-processed (B) with Quicktome^TM^ (Omniscient Neurotechnology, Haymarket, NSW, Australia). As part of this process, the subject’s brain is set to Montreal Neurologic Institute (MNI) space, parcellated, and sorted into 8 discrete networks (C). Next, the connectome is generated (D) as a matrix of parcel-to-parcel BOLD-time signal correlations (R), and then these R values are compared to normal controls to generate a matrix of abnormal connectivity (E, |R| > 2SD from control means). Subfigure D displays the correlation matrix of the left central executive network (CEN): each row and column of the correlation matrix (right) represents a parcel, and the color of each box represents the correlation (R) between each parcel pair. The scale ranges from strongly anticorrelated (−1, dark blue) to uncorrelated (0, white) to strongly correlated (1, dark red). The 3D MRI (left) displays the values from the highlighted column of the matrix in their anatomical positions with parcel a9-46v selected as the seed (yellow). Similarly, with the same seed selected, subfigure E displays the anomaly matrix (right) which highlights parcel pairs that are non-anomalous (R within 2 SD; white), hyper-anomalous (R > 2SD, red), or hypo-anomalous (R < 2SD, blue). The 3D MRI (left) displays the anomalous parcel count (purple scale) from the highlighted column of the matrix with parcel a9-46v selected as a seed.

Imaging data was then preprocessed using FDA-cleared clinical connectomic software (Quicktome^TM^, Omniscient Neurotechnology, Haymarket, NSW, Australia).^9^^,^^34–36^ Generally, the software strips the skull, motion corrects, repetition time (TR) censors, confound regresses, bandpass filters, and global signal regresses the images. Imaging data then gets registered into Montreal Neurological Institute (MNI) space, a coordinate-based brain template that standardizes patient neuroimaging, and parcellated according to the Glasser atlas.^10^ Parcel-level BOLD-time signals over the entire exam period are then compared across the entire brain to generate Pearson’s correlation coefficients (R), representing the functional connectome (Figure 2).

### Abnormal Functional Connectivity

Because neither high nor low connectivity between any two parcels is necessarily abnormal, we then used the software’s built-in anomaly-matrix function to compare each R value against a set of over 2000 healthy controls obtained from five different publicly available data banks with a broad age and balanced sex distribution.^37^ This calculation adjusts for highly similar (i.e., collinear) functional connectivity patterns,^20^ inherent low dimensionality,^38^^,^^39^ and collinearity among parcel connectivity patterns without prior dimensionality reduction.^40^ It then uses tangent space projection to identify values outside ±2 standard deviations (SD) from control means for each parcel pair corresponding to a two-tailed *P* value. Because each network varies in size, we then converted the total number of within-network anomalies into a percentage by dividing by the total number of possible abnormal connections, representing the proportion of connections that are abnormal within each of the four networks of interest (CEN, DMN, LANG, SN) (Supplementary Figure S1).

### Tumor Variables

Tumor variables included pathology, WHO grade, volume, laterality, O6-methylguanine-DNA methyltransferase [MGMT] methylation status, isocitrate dehydrogenase (IDH) status, and anatomical location [lobe] (Table 1). Tumor volume was measured via contrast enhancement on T1-weighted post-contrast MRIs for high grade gliomas and metastases. For low grade gliomas, size was measured using T2-FLAIR.

### Statistical Correlation Analysis

Spearman’s rank correlation coefficients (ρ) were calculated to assess relationships between percent-within network anomalies for the four networks of interest (CEN, DMN, LANG, and SN) and tumor variables (pathology, WHO grade, volume, laterality, MGMT methylation status, IDH status, and anatomical location [lobe]) with neuropsychological scores (TMT-B, COWAT, and WAIS-DS) using MATLAB (The MathWorks, Inc, Natick, Massachusetts). A Benjamini-Hochberg (BH) procedure was applied to control for false discovery rate (FDR) at a threshold of 0.05. Raw *P* values from the Spearman correlations were ranked in ascending order, and adjusted significance thresholds were computed using the BH formula. Correlations with *P* values less than 0.05 were considered significant.^41^

### Mediation Analysis

Next, exploratory mediation analyses were conducted in *R* (v4.5.1; R Core Team, 2025; Vienna, Austria) using the *lavaan* packages^42^ for variables with significant (1) tumor-to-connectivity and (2) connectivity-to-neuropsychological score correlations (Table 2). A square root transformation of anomaly data was performed as it was non-normally distributed (Shapiro-Wilk *P* = 0.002), and 95% confidence intervals (CI) were obtained from bootstrapping the model 5000 times. Mediation analyses generated raw and standardized β and 95% CIs. Standardized values were calculated by z-scoring the raw data before running the structural equation modeling (Supplementary Table S6). This analysis was also run with raw anomaly data, and it was also significant in the same manner (suggesting that this result was not a product of the square root transformation). Where applicable, continuous variables are displayed as mean ± SD.

**Table 2.** Summary of the significant correlations

Parameter	Correlation	rho; ρ	*P* value
Connectivity-Neuropsych			
	LesSN-WAIS-DS	0.53	0.006
	RSN-COWAT	0.50	0.012
	LesLANG-TMT-B	0.46	0.017
	RCEN-TMT-B	0.45	0.017
	LesSN-COWAT	0.45	0.017
	LesCEN-WAIS-DS	0.42	0.023
	BilatLANG-TMT-B	0.42	0.024
	RSN-WAIS-DS	0.42	0.023
Tumor-Neuropsych			
	Lat-COWAT	0.47	0.017

Salience network (SN), Central executive network (CEN), Lesional (Les), Right (R), Trail Making Test (Part B, TMT-B), Wechsler Adult Intelligence Scale (WAIS)-4 Digit Span (WAIS-DS), Controlled Oral Word Association Test (COWAT), Lesion laterality (Lat), Volume (Vol), and Neuropsychological Test Score (Neuropsych).

Rho represents Spearman’s rank correlation coefficients (rho; ρ).

## Results

### Demographic and Clinical Characteristics

Thirty males (58.8%) and 21 females (41.2%) were included in our study. The average age was 56.7 years (SD ± 14.29 years) and ranged from 25 to 83 years. Forty-eight patients were white, 1 was Asian, and 2 were black. Forty-four patients were right-handed (86.3%), and 7 patients were left-handed (13.7%). This cohort had a mean KPS score of 90 ± 10.14. The average time between neuropsychological tests and rs-fMRI exams was 3.47 ± 7.77 days, and the average time between neuropsychological tests and surgery was 8.75 ± 14.57 days. The average neuropsychological test z-score was −1.71 ± 2.08 for TMT-B, −0.253 ± 0.804 for WAIS-DS, and -0.66 ± 1.49 for COWAT. Of note, for all neuropsychological tests, higher z-scores were associated with better performance. Demographic, neuropsychological, and clinical variables for our cohort are listed in Table 1.

### Functional Connectivity Characteristics

Abnormal functional connectivity was analyzed within the four networks of interest, and the average percent of abnormal connections within each network was calculated within each hemisphere, bilaterally, and whether they were on the same (i.e., lesional) or opposite (i.e., contralesional) side as the brain tumor (Supplementary Table S2). We then examined which anatomical regions of the brain had the most abnormal connectivity for the relevant functional networks, and the results are presented in Supplementary Table S3.

### Abnormal Connectivity and Neuropsychological Performance

**Figure 3.**
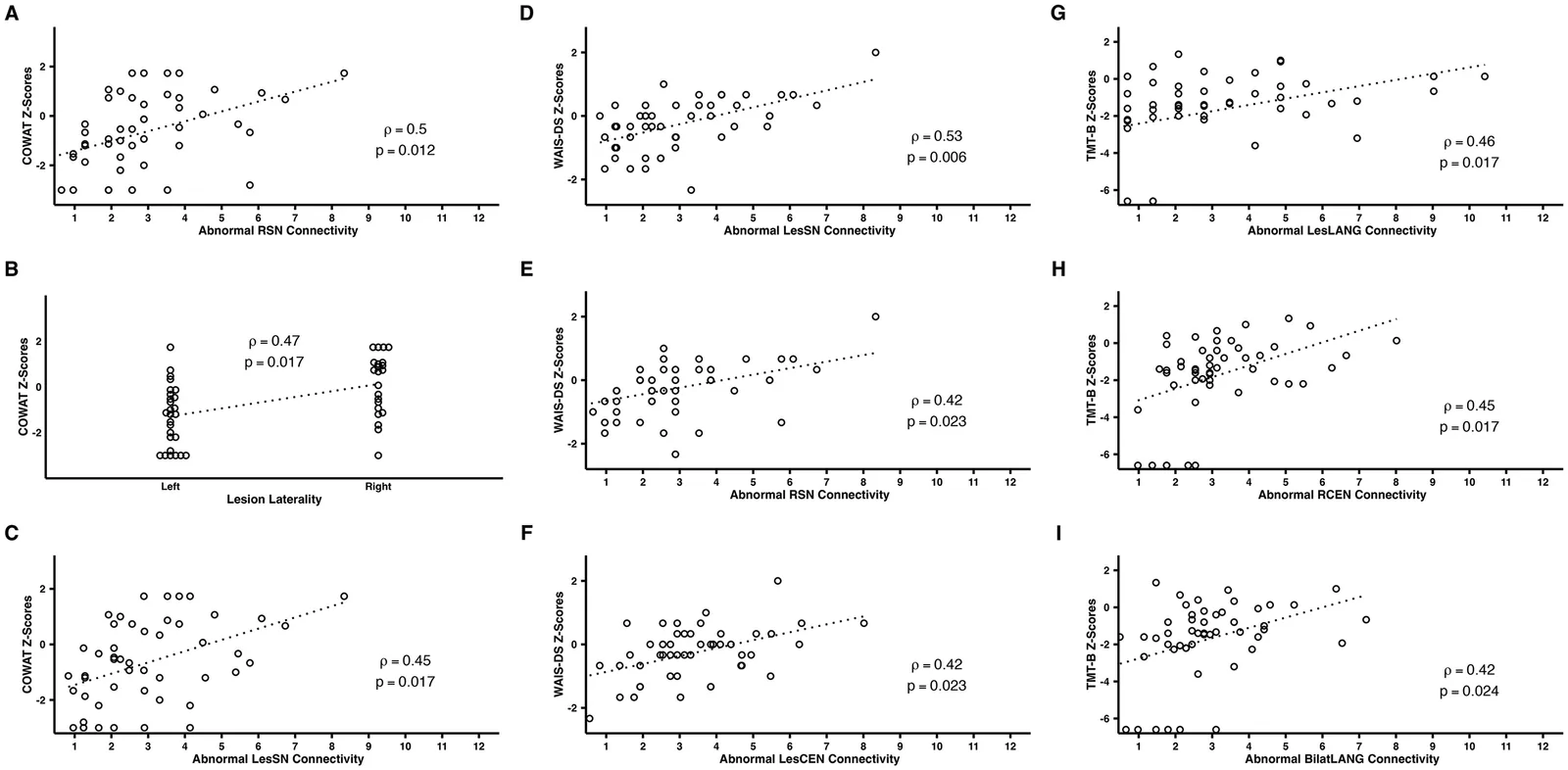
Significant correlations between neuropsychological tests, connectome anomalies, and tumor variables. Each data point represents an individual participant. Significant correlations included: COWAT performance to right-SN connectivity (A; *ρ* = 0.50, *P* = 0.012), lesion laterality (B; *ρ* =  0.47, *P* = 0.017), and lesional-SN connectivity (C; *ρ* = 0.45, *P* = 0.017); WAIS-DS performance to lesional-SN (D; *ρ* = 0.53, *P* = 0.006), right-SN (E; *ρ* = 0.42, *P* = 0.023), and lesional-CEN (F; *ρ* = 0.42, *P* = 0.023) connectivity; and TMT-B to lesional-LANG (G; *ρ* =  0.46, *P* = 0.017), right-CEN (H; *ρ* = 0.45, *P* = 0.017), and bilateral-LANG (I; *ρ* =  0.42, *P* = 0.024) connectivity.

Out of 81 total correlations, abnormal connectivity within 6 sub-networks significantly correlated with neuropsychological performance after FDR correction (Table 2 and Supplementary Table S4): lesional and right SN, lesional and right CEN, and lesional and bilateral LANG. Performances on WAIS-DS were significantly correlated with abnormal functional connectivity in the (1) lesional-SN (*ρ* = 0.53, *P* = 0.006), (2) lesional-CEN (*ρ* = 0.42, *P* = 0.023), and (3) right SN (*ρ* = 0.42, *P* = 0.023). Performances on COWAT were significantly correlated with abnormal functional connectivity in the (1) right SN (*ρ* = 0.50, *P* = 0.012) and (2) lesional-SN (*ρ* = 0.45, *P* = 0.017). Finally, performances on TMT-B were significantly correlated with abnormal functional connectivity in the (1) lesional-LANG (*ρ* =  0.46, *P* = 0.017), (2) right CEN (*ρ* = 0.45, *P* = 0.017), and (3) bilateral LANG (*ρ* =  0.42, *P* = 0.024). For all these relationships, more within-network anomalies were associated with better test performances (Figure 3).

### Tumor Variables and Neuropsychological Performance

Lesion laterality was significantly correlated with COWAT performance (*ρ* = 0.47, *P* = 0.017), such that having a right-sided tumor was associated with improved performance (Figure 3B and Supplementary Table S4). No other tumor variables (pathology, WHO grade, volume, MGMT status, IDH status, and anatomical location [lobe]) significantly correlated with neuropsychological test performance after FDR correction (Supplementary Table S4).

### Tumor Variables and Abnormal Network Connectivity

For the eight abnormal connectivity-to-neuropsychological performance correlations listed in Table 2, we conducted a *post hoc* analysis looking for associations between tumor variables and this subset of abnormal connectivity. Prior to FDR correction, lesion laterality was correlated with abnormal right CEN (*r* = 0.37, *P* = 0.008) and right SN (*r* = 0.30, *P* = 0.03) connectivity, and IDH-status was correlated with abnormal lesional-CEN connectivity (*r* = 0.31, *P* = 0.044). However, when FDR corrected, no tumor variable remained significantly correlated (Supplementary Table S5).

### Mediation Analyses

**Figure 4.**
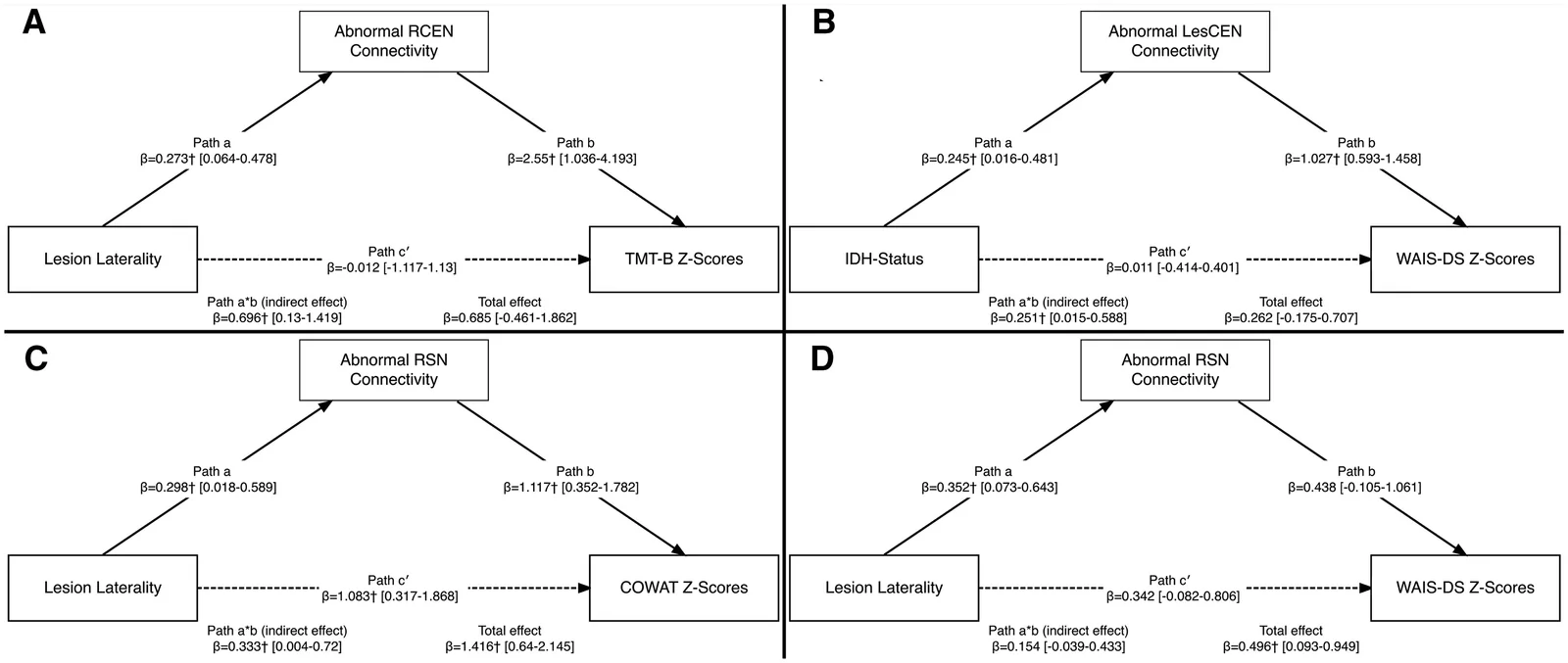
Changes in functional connectivity mediate the relationship between tumor variables and neuropsychological performance. (A) Abnormal right CEN connectivity fully mediated the relationship between lesion laterality and TMT-B performance. (B) Abnormal lesional-sided CEN connectivity fully mediated the relationship between IDH-status and WAIS-DS performance. (C) The relationship between lesion laterality and COWAT was partially mediated through abnormal right SN connectivity. (D) The influence of lesion laterality on WAIS-DS performance was not mediated by right SN connectivity. Path a reflects the relationship between the tumor variable and connectivity metric (mediator). Path b reflects the relationship between connectivity and neuropsychological test performance. Path c’ reflects the direct relationship between the tumor variable and neuropsychological test score when the indirect effect (tumor variable to connectivity to performance) is removed. Path a*b (indirect effect) reflects the influence tumor variables have on neuropsychological performance via connectivity. Path c’+a*b (total effect) reflects the combined relationship between tumor variables and neuropsychological performance when both the direct effect and indirect effect via connectivity are included. β (beta) represents the raw regression coefficient (i.e., the estimated effect size of one variable on another within the regression model), and the 95% confidence intervals of the raw regression coefficients are included in brackets for each path. Significance was determined whether the 95% CI from the bootstrapped models involved 0. See Supplementary Table S6 for standardized β and 95% CI values.

We then performed exploratory, statistical mediation analyses focused on the relationships identified above with the highest correlations for (1) tumor-to-connectivity and (2) connectivity-to-neuropsychological test scores (Figure 4).

#### Lesion Laterality, Right Central Executive Network Connectivity, and TMT-B Scores

Abnormal right CEN connectivity mediated the relationship between lesion laterality and TMT-B performance (Figure 4A and Supplementary Table S6). Lesion laterality was significantly associated with abnormal right CEN connectivity (path a; *β* = 0.273 [0.064, 0.478]) such that right-sided lesions were associated with greater abnormal right CEN connectivity. Abnormal right CEN connectivity was significantly associated with TMT-B z-scores (path b; *β* = 2.55 [1.036, 4.193]) such that greater abnormal right CEN connectivity was associated with better TMT-B performance. The indirect effect was significant (path a*b; *β* = 0.696 [0.13, 1.419]), indicating that the influence of lesion laterality on TMT-B performance was mediated by abnormal functional connectivity within the right CEN. The direct effect was not significant (path c’; *β* = −0.012 [−1.117, 1.13]), suggesting that once the influence of abnormal right CEN connectivity was removed from the model, lesion laterality was no longer independently correlated with TMT-B performance. Finally, the total effect was not significant (path c’+a*b; β = 0.685 [−0.461, 1.862]), indicating that the relationship between lesion laterality and TMT-B performance was fully mediated by abnormal right CEN connectivity.

#### IDH-Status, Lesional Central Executive Network Connectivity, and WAIS-DS Scores

Abnormal lesional-CEN connectivity mediated the relationship between IDH-status and WAIS-DS performance (Figure 4B and Supplementary Table S6). IDH-status was significantly associated with abnormal lesional-CEN connectivity (path a; *β* = 0.245 [0.016, 0.481]) such that having an IDH-mutant glioma was associated with greater abnormal lesional-CEN connectivity. Abnormal lesional-CEN connectivity was significantly associated with WAIS-DS z-scores (path b; *β* = 1.027 [0.593, 1.458]) such that greater abnormal lesional-CEN connectivity was associated with better WAIS-DS performance. The indirect effect was significant (path a*b; *β* = 0.251 [0.015, 0.588]), indicating that the influence of IDH-status on WAIS-DS performance was mediated via abnormal functional connectivity within the lesional-CEN. The direct effect was not significant (path c’; *β* = 0.011 [−0.414, 0.401]), suggesting that once the influence of lesional-CEN connectivity was removed from the model, IDH-status was no longer independently correlated with WAIS-DS performance. Finally, the total effect was not significant (path c’+a*b; *β* = 0.262 [−0.175, 0.707]), indicating that the relationship between IDH-status and WAIS-DS performance was fully mediated by abnormal lesional-CEN connectivity.

#### Lesion Laterality, Right Salience Network Connectivity, and COWAT Scores

The relationship between lesion laterality and COWAT was partially mediated through abnormal right SN connectivity (Figure 4C and Supplementary Table S6). Lesion laterality was significantly associated with abnormal right SN connectivity (path a; *β* = 0.298 [0.018, 0.589]) such that more right-sided lesions were associated with greater abnormal right SN connectivity. Abnormal right SN connectivity was significantly associated with COWAT z-scores (path b; *β* = 1.117 [0.352, 1.782]) such that greater abnormal right SN connectivity was associated with better COWAT performance. The relationship between lesion laterality and COWAT was significant both when right SN connectivity was included in the analysis (indirect pathway; path a*b; *β* = 0.333 [0.004, 0.720]) and removed (direct pathway; path c’; *β* = 1.083 [0.317, 1.868]), suggesting a partial mediation effect on the influence between lesion laterality and COWAT performance. Right-sided tumors were associated with an increase in abnormal right SN connectivity, and, directly and indirectly, better performance on COWAT. Finally, the total effect was significant (path c’+a*b; *β* = 1.416 [0.64, 2.145]), confirming the partial mediation effect and supporting our findings from earlier that lesion laterality and COWAT were significantly correlated (*ρ* = 0.47, *P* =0.017).

#### Lesion Laterality, Right Salience Network Connectivity, and WAIS-DS Scores

We were unable to detect a mediation effect between lesion laterality, right SN connectivity, and WAIS-DS performance, likely due to underpowering (Figure 4D and Supplementary Table S6). Lesion laterality and WAIS-DS performance (total effect; path c’+a*b; *β* = 0.496 [0.093, 0.949]) and lesion laterality and right SN connectivity (path a; *β* = 0.352 [0.073, 0.643]) were significantly correlated; however, the influence of lesion laterality on WAIS-DS performance was not mediated by right SN connectivity (path a*b; *β* = 0.154 [−0.039, 0.433]), and the relationship between lesion laterality and WAIS-DS performance became non-significant when right SN connectivity (path c’; *β* = 0.342 [−0.082, 0.806]) was introduced to the model. Right SN connectivity was not significantly correlated with WAIS-DS performance in this model (path b; *β* = 0.438 [−0.105, 1.061]). Given the total effect was significant, lesion laterality and WAIS-DS may be correlated, but our study was too underpowered to detect the individual relationships (path b, c’, a*b) that mediate this correlation.

## Discussion

Here we investigated the relationships between abnormal functional brain network connectivity and neuropsychological testing in 51 consecutive people with intra-axial brain tumors. Altogether, in each functional network, more anomalies were associated with better performance regardless of cognitive domain, suggesting compensatory—rather than pathological—adaptations may be responsible for some of the clinical phenotype heterogeneity seen across otherwise similar anatomical locations and oncological pathologies.

### Salience Network and Attention-and-Semantics

Greater abnormal functional connectivity within the right and lesional-SN was associated with improved cognitive performance on tests that assess attentional control, distractibility, concentration, semantics, and working memory (COWAT and WAIS-DS).^29^ As a unique contribution to this existing literature, our regional-level anomaly data identified highly anomalous areas within the SN that appeared to drive these significant correlations. In our data within the lesional-SN, the left insula and left supramarginal gyrus showed the greatest degree of abnormal connectivity. The left insula plays a role in coordinating speech articulation,^43^ and the left supramarginal gyrus is active while holding a sequence of numbers in working memory.^44^ Similarly, within the right SN, circuits within the anterior half of the precentral gyrus, pars opercularis, and middle cingulate gyrus showed the greatest degree of abnormal connectivity. The involved premotor area has been shown to be important for oral/motor sequencing in digit recitation,^45^ and regions such as the middle cingulate gyrus are involved in verbal working memory.^46^ Therefore, increased abnormal connectivity in these regions in patients who have better neuropsychological test scores may reflect a compensatory functional rewiring that allows for the relative preservation of these functions in better performing cases.

### Central Executive Network and Executive Function

More right-sided CEN anomalies were associated with better performance on executive function and concentration tests. Furthermore, the influence of lesion laterality on TMT-B performance was fully mediated by abnormal right-sided CEN connectivity (Figure 4A). This is also consistent with previous literature demonstrating that stimulus-driven shifts of spatial attention and target detection are integral to completing the TMT-B, and they are largely managed by the right hemispheric portion of the CEN.^47^ Our regional-level anomaly data demonstrated that anomalies within the right CEN were concentrated within the inferior frontal sulcus, posterior middle temporal gyrus, and posterior medial middle frontal gyrus. These association cortices are known to be important in tasks that require visual working memory (i.e., remembering to switch between numbers and letters during TMT-B) by (1) creating procedural representations from verbal instructions and (2) maintaining where objects are in space to assist in motor planning.^48^ The anterior insula was also highly abnormal within our population, and it has been shown to play a role in interpreting sensory information and choosing an action or judgment based on that input, as well as time perception (TMT-B is a timed task).^48^ Overall, greater abnormal connectivity within the right CEN—mainly, the inferior frontal sulcus, posterior middle temporal gyrus, posterior medial middle frontal gyrus, and the anterior insula—was associated with better performance on executive function and concentration-based cognitive tasks, again suggesting that some degree of compensatory functional reconnectivity may be responsible for maintaining performance.

### Central Executive Network and Attention

More lesional-CEN anomalies were associated with better performance on the WAIS-DS—a task that measures attention. We found the influence of IDH-status on WAIS-DS performance was fully mediated by abnormal lesional-CEN connectivity. The relationship was such that having an IDH-mutant glioma was associated with improved WAIS-DS performance via an increase in abnormal lesional-CEN functional connectivity (Figure 4B). This relationship—between IDH-status and WAIS-DS performance—is consistent with existing literature,^49^ and our study adds a novel addition to this relationship by uncovering the mediation effect of abnormal CEN functional connectivity. Furthermore, we found that specific regions of the CEN—mainly the left supramarginal gyrus, left posterior cingulate, and left middle and inferior temporal gyri— demonstrated the greatest amount of connectivity compensation. We previously mentioned how the left supramarginal gyrus is important during WAIS-DS (holding a sequence of numbers in working memory), and, while the involvement of the left posterior cingulate cortex and left middle and inferior gyri have been less explored in WAIS-DS tasks, they are shared regions across the CEN and DMN. Reciprocal connections between these regions—and networks—has been shown to predict working memory performance.^50^ Furthermore, IDH-mutant gliomas are known to grow more slowly than IDH-wild type lesions, potentially allowing for more time for compensatory functional rewiring to maintain function. Slower lesional speed is a known factor in allowing for more functional-anatomical reorganization, which is consistent with this finding.^51–53^

### Language Network and Executive Function

More lesional and bilateral LANG anomalies were associated with better performance on executive function and concentration tests. Of note, our cohort was predominantly right-handed (44/51) and had more left sided tumors (28). For both the lesional and bilateral LANG, networks, regions that exhibited the highest levels of abnormal connectivity were within the same regions of the right hemisphere (superior temporal gyrus and sulcus, temporopolar region, and middle temporal gyrus). We hypothesize that, because the TMT-B requires participants to use a pencil and alternate between letters and numbers, it requires high-level social and perceptual processing like interpreting hand motion and tool use, which involve aspects of the superior and middle temporal cortices. Therefore, given these known anatomical-functional relationships, the remapping of these regions may relate directly to improved performance on the TMT-B.^54^

### Lesion Laterality and COWAT

There was a significant correlation between lesion laterality and COWAT scores, such that having a right-sided tumor was associated with improved performance. The COWAT requires participants to orally list words that start with a specific letter (Supplementary Table S1). This correlation was unsurprising given the language component of the exam, that a significant majority of our cohort was right-handed (44 of 51), and that left-hemisphere language lateralization is dominant in 95% of right-handed individuals.^55^ Notably, we did not find any other significant correlations between tumor variables and neuropsychological test scores before or after FDR correction.

### Default Mode Network

Similar to previous studies,^56^ we did not find any significant correlations between neuropsychological test scores and abnormal DMN connectivity. As explored earlier, the DMN is tightly associated with the SN, is most active during resting states, and is associated with self-directed (i.e., “internal”) thoughts.^25^ We hypothesize that the lack of correlation with the DMN may be due to the chosen tasks not directly reflecting isolated DMN activity, tumor locations not predominantly involving the DMN, lack of compensatory capacity within the DMN, and/or our study being too underpowered to detect such correlations.

### Limitations and Future Directions

This study examined correlations between abnormal within-network functional connectivity and neuropsychological test scores in people with brain tumors prior to surgery. While this study established statistically significant correlations, it does not establish causality, nor does it definitively differentiate pathological from compensatory abnormalities. Additionally, our analysis was limited to within-network connectivity, and, therefore, may not capture critical between-network dynamics. Furthermore, this study does not account for, nor assess, the effects of baseline demographics, steroid use, behavioral interventions, medications, psychological stressors, or other non-tumor-related potential confounders. It further does not differentiate between individual baseline neuropsychological performance (which is largely unknown) and any new deficit caused by the brain tumor (however, neuropsychological test scores are normalized to published age- and education-matched norms to minimize the impact of this confounder). Finally, this study only focused on the preoperative setting. Future work will include longitudinal and postoperative analyses of functional connectivity with the goal of informing patient risk stratification, guiding individualized surgical planning, and identifying potential neuromodulation targets for stimulation-assisted neurocognitive rehabilitation.

## Conclusion

In people with brain tumors, neuropsychological performance within three domains (attention, attention-and-semantics, and executive function) correlated with abnormal functional connectivity more than classical anatomical and tumor variables. This finding, together with exploratory mediation analyses, provides preliminary support for the theory that compensatory functional connectivity may at least partially explain the heterogeneity of neuropsychological presentations seen in people with otherwise similar intra-axial brain tumors. Future work will investigate between-network relationships, as well as incorporate prospective, longitudinal analyses with normative controls to better establish causality, isolate the organic contributions of brain tumors, and assess the impact of surgical, radiation, and medical interventions.

## Supplementary Material

vdag084_Supplementary_Data

## Data Availability

Data from this study will be made available upon reasonable request.
