# Correction to: Exploring relationships between abnormal within-network functional connectivity, preoperative brain tumor variables, and neuropsychological test scores

**DOI:** 10.1093/noajnl/vdag227

**Published:** 2026-09-08

This is a correction to: Bryce J Laurin, Randall Treffy, Christina Feller, Joshua Wilder, Krish Vasudev, Nicholas Schulz, Léon Taquet, Melissa Lancaster, Alissa Butts, Timothy F Boerger, Brian D Schmit, Max O Krucoff, Exploring relationships between abnormal within-network functional connectivity, preo­perative brain tumor variables, and neuropsychological test scores, *Neuro-Oncology Advances*, Volume 8, Issue 1, January-December 2026, vdag084, https://doi.org/10.1093/noajnl/vdag084

In the originally published version of this manuscript, author name Nicholas Schulz was misspelled.

This error has been corrected.

