# Supplementary material for: Exploring relationships between abnormal within-network functional connectivity, preoperative brain tumor variables, and neuropsychological test scores
Source: Neurooncol Adv. 2026 Apr 3;8(1):vdag084. doi: 10.1093/noajnl/vdag084 (PMC13157344; doi:10.1093/noajnl/vdag084)
Supplement: vdag084_Supplementary_Data [file vdag084_supplementary_data.zip › NOAR1_Supplementary1_Final.docx]

| Domain | Test Name | Test Task | Test Measures | Score |
| --- | --- | --- | --- | --- |
| Executive Function | Trail Making Test-B | Ordering alternating numbers and letters | Cognitive flexibility, Sequencing,  problem solving | Time required to complete |
| Attention | Wechsler Adult Intelligence  Scale (WAIS)-4 Digit Span | Repeat numbers in order from smallest to largest | Working memory manipulation,  Distractibility | Number of correctly repeated sequences |
| Attention & Semantics | Controlled Oral Word Association Test | Words that start with | Verbal fluency, Attention & semantics | Total number of valid words |

Supplementary Table 1. Neuropsychological Battery Test Specifics. Tests used for this study, their directions, and how they are scored.

|  | Central Executive Network | | Language Network | | Salience Network | |
| --- | --- | --- | --- | --- | --- | --- |
|  | Mean %  anomalies | SD | Mean %  anomalies | SD | Mean %  anomalies | SD |
| Bilateral Total | 3.03 | 1.11 | 2.87 | 1.41 | 2.55 | 0.87 |
| Left Total | 3.10 | 1.45 | 2.97 | 1.92 | 2.33 | 1.28 |
| Right Total | 3.15 | 1.47 | 2.75 | 2.20 | 2.90 | 1.71 |
| Lesional Total | 3.46 | 1.50 | 3.06 | 2.41 | 3.02 | 1.65 |
| Contralesional Total | 2.80 | 1.35 | 2.66 | 1.63 | 2.21 | 1.29 |

Supplementary Table 2. Network-Level Anomalies. Summary table demonstrating average percent anomalous network, broken down by network and anomaly location for the CEN, LANG, and SN networks. SD, standard deviation.

| Network | Parcel Name | Mean % abnormal  connections |
| --- | --- | --- |
| LesCEN | L_TE1p | 4.83 |
|  | L_TE2a | 4.14 |
|  | L_RSC | 3.78 |
|  | L_PFm | 3.77 |
| LesSN | L_MI | 3.48 |
|  | L_PFcm | 3.42 |
|  | L_PI | 3.40 |
|  | L_PF | 3.36 |
| LesLANG | R_STSvp | 6.93 |
|  | R_TGd | 5.55 |
|  | R_TE1a | 5.39 |
|  | R_STSdp | 5.23 |
| RCEN | R_IFSp | 3.93 |
|  | R_TE1p | 3.76 |
|  | R_AVI | 3.65 |
|  | R_i6-8 | 3.58 |
| RSN | R_a24pr | 3.87 |
|  | R_55b | 3.77 |
|  | R_5mv | 3.58 |
|  | R_FOP1 | 3.56 |
| BilatLANG | R_STSvp | 5.77 |
|  | R_TGd | 4.41 |
|  | R_STSdp | 4.18 |
|  | R_STSda | 4.00 |

Supplementary Table 3. Average (mean) percent anomalies by brain parcel across correlated networks: lesional CEN, LANG, and SN, right CEN and SN, and bilateral LANG. As an exploratory, observational analysis, the four parcels that exhibited the greatest number of abnormal connections are included here.

| Tumor or Connectivity Variable | Neuropsych Test | Spearman rho | Raw p-value | FDR-corrected p-value |
| --- | --- | --- | --- | --- |
| SALLes | DSSeq | 0.530 | 0.000075 | 0.006 |
| SALRight | COWAT | 0.495 | 0.000297 | 0.012 |
| Lat | COWAT | 0.471 | 0.001 | 0.017 |
| LANGLes | TMTB | 0.458 | 0.001 | 0.017 |
| CENRight | TMTB | 0.448 | 0.001 | 0.018 |
| SALLes | COWAT | 0.448 | 0.001 | 0.017 |
| SALRight | DSSeq | 0.425 | 0.002 | 0.024 |
| CENLes | DSSeq | 0.421 | 0.002 | 0.023 |
| LANGBilateral | TMTB | 0.416 | 0.003 | 0.024 |
| Grade | COWAT | -0.420 | 0.006 | 0.051 |
| SALLes | TMTB | 0.363 | 0.010 | 0.071 |
| LANGRight | TMTB | 0.362 | 0.010 | 0.066 |
| CENLes | TMTB | 0.360 | 0.010 | 0.064 |
| CENBilateral | DSSeq | 0.357 | 0.011 | 0.064 |
| CENRight | COWAT | 0.351 | 0.013 | 0.072 |
| CENRight | DSSeq | 0.335 | 0.017 | 0.088 |
| DMNRight | TMTB | 0.319 | 0.024 | 0.115 |
| Grade | DSSeq | -0.333 | 0.029 | 0.131 |
| SALBilateral | DSSeq | 0.308 | 0.029 | 0.125 |
| DMNRight | DSSeq | 0.306 | 0.031 | 0.125 |
| LANGLes | DSSeq | 0.302 | 0.033 | 0.127 |
| CENBilateral | TMTB | 0.295 | 0.038 | 0.138 |
| LANGRight | DSSeq | 0.294 | 0.038 | 0.135 |
| LANGLeft | TMTB | 0.293 | 0.039 | 0.131 |
| LANGBilateral | DSSeq | 0.291 | 0.040 | 0.131 |
| SALBilateral | TMTB | 0.290 | 0.041 | 0.127 |
| SALRight | TMTB | 0.281 | 0.048 | 0.145 |
| Lat | DSSeq | 0.278 | 0.051 | 0.146 |
| Path | DSSeq | -0.273 | 0.058 | 0.161 |
| Grade | TMTB | -0.290 | 0.059 | 0.159 |
| DMNContrales | DSSeq | 0.261 | 0.067 | 0.176 |
| DMNBilateral | DSSeq | 0.245 | 0.086 | 0.219 |
| CENLeft | COWAT | -0.219 | 0.130 | 0.319 |
| IDH | COWAT | 0.241 | 0.134 | 0.319 |
| DMNLes | DSSeq | 0.214 | 0.135 | 0.313 |
| LANGContrales | TMTB | 0.192 | 0.183 | 0.411 |
| DMNBilateral | TMTB | 0.188 | 0.191 | 0.419 |
| MGMT | TMTB | 0.198 | 0.208 | 0.443 |
| DMNLeft | TMTB | -0.177 | 0.218 | 0.453 |
| LANGContrales | DSSeq | 0.170 | 0.237 | 0.480 |
| DMNLeft | DSSeq | 0.169 | 0.242 | 0.478 |
| LANGLeft | DSSeq | 0.166 | 0.248 | 0.479 |
| SALBilateral | COWAT | 0.167 | 0.252 | 0.475 |
| Lat | TMTB | 0.159 | 0.271 | 0.499 |
| CENLes | COWAT | 0.156 | 0.283 | 0.510 |
| DMNContrales | TMTB | 0.154 | 0.287 | 0.505 |
| MGMT | DSSeq | 0.168 | 0.287 | 0.495 |
| Path | COWAT | -0.150 | 0.310 | 0.523 |
| IDH | DSSeq | 0.160 | 0.311 | 0.513 |
| Loc | COWAT | 0.148 | 0.311 | 0.503 |
| MGMT | COWAT | 0.148 | 0.361 | 0.573 |
| CENLeft | DSSeq | 0.132 | 0.361 | 0.562 |
| DMNLes | COWAT | 0.122 | 0.405 | 0.620 |
| LANGLes | COWAT | 0.113 | 0.438 | 0.657 |
| CENBilateral | COWAT | 0.103 | 0.480 | 0.707 |
| CENContrales | TMTB | 0.101 | 0.486 | 0.702 |
| CENContrales | COWAT | -0.101 | 0.492 | 0.699 |
| Loc | IDH | 0.098 | 0.535 | 0.747 |
| Vol | DSSeq | 0.092 | 0.536 | 0.736 |
| Vol | COWAT | -0.085 | 0.570 | 0.770 |
| IDH | TMTB | 0.088 | 0.581 | 0.771 |
| DMNLes | TMTB | 0.080 | 0.581 | 0.759 |
| CENLeft | TMTB | 0.075 | 0.603 | 0.775 |
| SALContrales | DSSeq | -0.075 | 0.605 | 0.766 |
| Path | TMTB | 0.066 | 0.652 | 0.812 |
| SALLeft | TMTB | 0.040 | 0.781 | 0.959 |
| LANGContrales | COWAT | -0.040 | 0.785 | 0.949 |
| DMNRight | COWAT | 0.040 | 0.787 | 0.937 |
| DMNContrales | COWAT | -0.040 | 0.787 | 0.924 |
| Loc | TMTB | -0.039 | 0.790 | 0.914 |
| SALLeft | COWAT | -0.039 | 0.792 | 0.903 |
| LANGLeft | COWAT | 0.037 | 0.799 | 0.899 |
| DMNLeft | COWAT | 0.036 | 0.809 | 0.897 |
| LANGBilateral | COWAT | -0.033 | 0.823 | 0.900 |
| SALLeft | DSSeq | -0.022 | 0.881 | 0.951 |
| LANGRight | COWAT | 0.022 | 0.883 | 0.941 |
| Loc | DSSeq | -0.015 | 0.919 | 0.967 |
| SALContrales | COWAT | 0.011 | 0.938 | 0.974 |
| DMNBilateral | COWAT | -0.008 | 0.955 | 0.979 |
| Vol | TMTB | 0.008 | 0.957 | 0.969 |
| CENContrales | DSSeq | 0.007 | 0.960 | 0.960 |
| SALContrales | TMTB | -0.007 | 0.963 | 0.952 |

Supplementary Table 4. Results of Spearman’s rank correlations. All 81 correlations demonstrating the relationship between tumor variables, connectivity metrics, and neuropsychological test, sorted by non-FDR-corrected p-values.

| Tumor Variable | Connectivity Variable | Spearman rho | Raw p-value | FDR-corrected p-value |
| --- | --- | --- | --- | --- |
| Lat | CENRight | 0.365 | 0.008 | 0.294 |
| Lat | SALRight | 0.298 | 0.033 | 0.586 |
| IDH | CENLes | 0.313 | 0.044 | 0.510 |
| Lat | SALLes | 0.255 | 0.071 | 0.623 |
| Grade | CENLes | -0.264 | 0.088 | 0.615 |
| MGMT | CENLes | 0.254 | 0.104 | 0.608 |
| Vol | SALRight | 0.230 | 0.112 | 0.559 |
| MGMT | SALRight | 0.233 | 0.137 | 0.601 |
| Grade | CENRight | -0.220 | 0.157 | 0.609 |
| Loc | LANGLes | -0.197 | 0.165 | 0.578 |
| MGMT | CENRight | 0.205 | 0.193 | 0.615 |
| MGMT | LANGLes | 0.203 | 0.197 | 0.574 |
| IDH | CENRight | 0.197 | 0.212 | 0.571 |
| Grade | SALLes | -0.181 | 0.246 | 0.614 |
| Path | LANGLes | 0.164 | 0.256 | 0.598 |
| IDH | SALRight | 0.176 | 0.265 | 0.581 |
| Path | SALRight | -0.159 | 0.269 | 0.554 |
| Grade | SALRight | -0.149 | 0.341 | 0.663 |
| Path | SALLes | -0.136 | 0.345 | 0.636 |
| IDH | SALLes | 0.146 | 0.356 | 0.623 |
| Lat | CENLes | 0.131 | 0.358 | 0.597 |
| MGMT | SALLes | 0.108 | 0.495 | 0.788 |
| Vol | LANGLes | 0.099 | 0.499 | 0.759 |
| Lat | LANGLes | -0.094 | 0.510 | 0.744 |
| Vol | CENLes | 0.096 | 0.512 | 0.717 |
| Path | CENLes | -0.093 | 0.522 | 0.703 |
| Grade | LANGLes | -0.083 | 0.595 | 0.771 |
| Loc | CENLes | -0.075 | 0.600 | 0.750 |
| Loc | SALRight | 0.072 | 0.615 | 0.743 |
| Loc | CENRight | 0.068 | 0.637 | 0.743 |
| Loc | SALLes | 0.054 | 0.704 | 0.795 |
| IDH | LANGLes | 0.046 | 0.772 | 0.844 |
| Vol | CENRight | 0.024 | 0.872 | 0.924 |
| Vol | SALLes | 0.020 | 0.893 | 0.919 |
| Path | CENRight | 0.004 | 0.979 | 0.979 |

Supplementary Table 5. Post hoc, exploratory analysis between tumor variables and connectivity metrics, sorted by non-FDR-corrected p-values.

| Parameter | Formula | raw β | raw 95%CI | Significance | std β | std 95%CI | Significance |
| --- | --- | --- | --- | --- | --- | --- | --- |
| **a-paths** |  |  |  |  |  |  |  |
|  | Lat-RCEN | 0.273 | [0.064, 0.478] | * | 0.346 | [0.083, 0.615] | * |
|  | IDH-LesCEN | 0.245 | [0.016, 0.481] | * | 0.268 | [0.012, 0.529] | * |
|  | Lat-RSN | 0.352 | [0.073, 0.643] | * | 0.33 | [0.069, 0.594] | * |
|  | Lat-RSN | 0.298 | [0.018, 0.589] | * | 0.293 | [0.033, 0.571] | * |
| **b-paths** |  |  |  |  |  |  |  |
|  | RCEN-TMT-B | 2.55 | [1.036, 4.193] | * | 0.482 | [0.197, 0.795] | * |
|  | LesCEN-WAIS-DS | 1.027 | [0.593, 1.458] | * | 0.593 | [0.34, 0.849] | * |
|  | RSN-WAIS-DS | 0.438 | [-0.105, 1.061] | NS | 0.289 | [-0.066, 0.696] | NS |
|  | RSN-COWAT | 1.117 | [0.352, 1.782] | * | 0.379 | [0.121, 0.608] | * |
| **c'-paths** |  |  |  |  |  |  |  |
|  | Lat-TMT-B | -0.012 | [-1.117, 1.13] | NS | -0.003 | [-0.273, 0.269] | NS |
|  | IDH-WAIS-DS | 0.011 | [-0.414, 0.401] | NS | 0.007 | [-0.262, 0.248] | NS |
|  | Lat-WAIS-DS | 0.342 | [-0.082, 0.806] | NS | 0.212 | [-0.058, 0.491] | NS |
|  | Lat-COWAT | 1.083 | [0.317, 1.868] | * | 0.362 | [0.113, 0.628] | * |
| **indirect effects** |  |  |  |  |  |  |  |
|  | Lat-RCEN-TMT-B | 0.696 | [0.13, 1.419] | * | 0.167 | [0.034, 0.34] | * |
|  | IDH-LesCEN-WAIS-DS | 0.251 | [0.015, 0.588] | * | 0.159 | [0.006, 0.366] | * |
|  | Lat-RSN-WAIS-DS | 0.154 | [-0.039, 0.433] | NS | 0.095 | [-0.024, 0.274] | NS |
|  | Lat-RSN-COWAT | 0.333 | [0.004, 0.72] | * | 0.111 | [0.005, 0.238] | * |
| **total effects** |  |  |  |  |  |  |  |
|  | Lat-RCEN-TMT-B | 0.685 | [-0.461, 1.862] | NS | 0.164 | [-0.118, 0.442] | NS |
|  | IDH-LesCEN-WAIS-DS | 0.262 | [-0.175, 0.707] | NS | 0.166 | [-0.122, 0.439] | NS |
|  | Lat-RSN-WAIS-DS | 0.496 | [0.093, 0.949] | * | 0.307 | [0.051, 0.578] | * |
|  | Lat-RSN-COWAT | 1.416 | [0.64, 2.145] | * | 0.473 | [0.22, 0.724] | * |

Supplementary Table 6. Mediation analyses for relationships with significant tumor to connectivity relationships within the significant connectivity to score variables. Raw and standardized (std) regression coefficients (β) with 95% confidence intervals (CIs) are reported for each pathway. a-paths represent associations between tumor variables and network anomalies; b-paths represent associations between network anomalies and z-scores on neuropsychological tests; c′-paths represent direct effects of tumor variables on performance after accounting for mediators. Indirect effects reflect the product of corresponding a- and b-paths. Each pathway is maintained by row. Significance was determined using 95% confidence intervals that did not include zero. Asterisks (*) denote statistically significant effects; NS indicates non-significant effects.


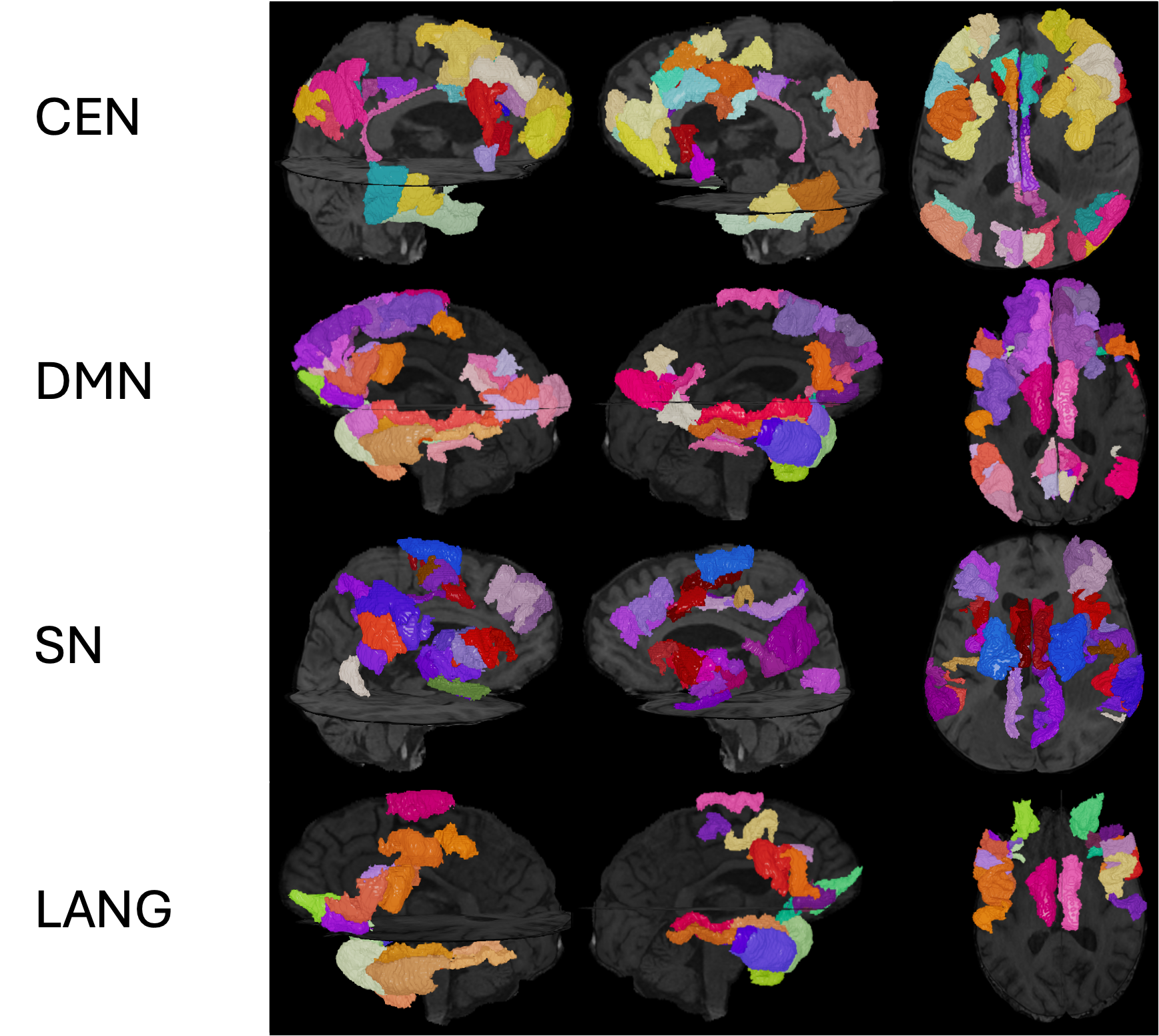


Supplementary Figure 1. Anatomical locations of parcels within the networks included in this study (central executive, default mode, salience, and language networks).
