# Supplementary figures and images for: Exploring relationships between abnormal within-network functional connectivity, preoperative brain tumor variables, and neuropsychological test scores
Source: Neurooncol Adv. 2026 Apr 3;8(1):vdag084. doi: 10.1093/noajnl/vdag084 (PMC13157344; doi:10.1093/noajnl/vdag084)

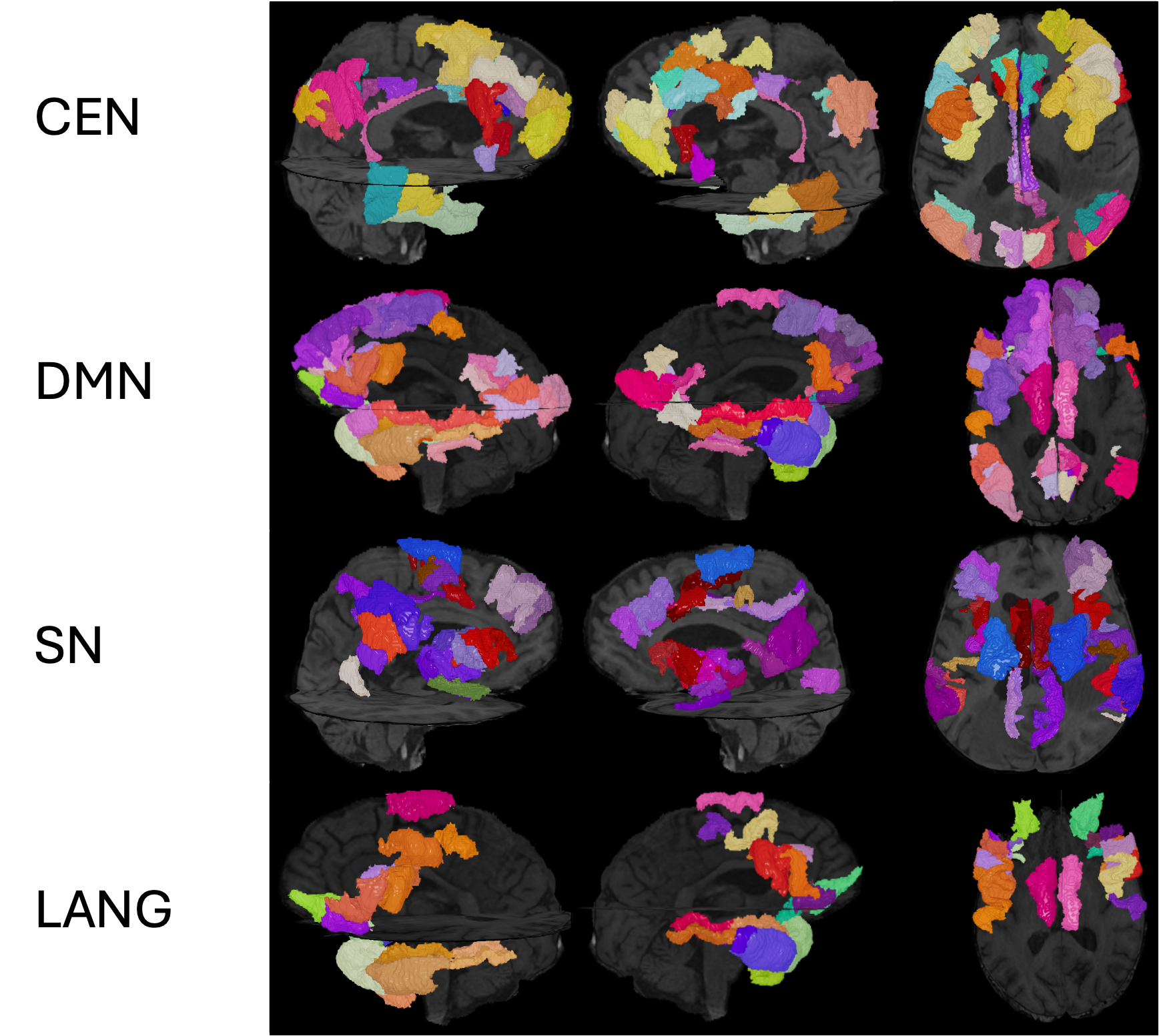

Supplement: vdag084_Supplementary_Data [file vdag084_supplementary_data.zip › SupplementaryFigure1.tiff]
